# SNAIL Induces EMT and Lung Metastasis of Tumours Secreting CXCL2 to Promote the Invasion of M2-Type Immunosuppressed Macrophages in Colorectal Cancer

**DOI:** 10.7150/ijbs.66854

**Published:** 2022-04-11

**Authors:** Zhen Bao, Wei Zeng, Di Zhang, Liyong Wang, Xin Deng, Jianqin Lai, Jian Li, Jin Gong, Guoan Xiang

**Affiliations:** 1 The Second School of Clinical Medicine, Southern Medical University, Guangzhou, Guangdong 510515, P.R. China.; 2 Department of General Surgery, Guangdong Second Provincial General Hospital, Guangzhou, Guangdong 510317, P.R. China.; 3 Department of General Surgery, The First Affiliated Hospital of Jinan University, Guangzhou, Guangdong 510632, P.R. China.; 4 Department of General Surgery, Yiyang Central Hospital, Yiyang, Hunan 413000, P.R. China.; 5 Department of General Surgery, Henan Cancer Hospital, Zhengzhou Henan, 450003, P.R. China.

**Keywords:** SNAIL, EMT, Lung Metastasis, CXCL2, Macrophages, Colorectal Cancer

## Abstract

**Background:** There is increasing evidence that tumour-associated macrophages (TAMs) are critical in the formation of lung metastases. However, the molecular mechanisms of tumour interactions with TAMs via EMT are largely unknown.

**Methods:** The mechanism of lung metastasis was studied in patient tissues. The mechanism of SNAIL regulation of the interaction between mesenchymal cells and M2 macrophages was elucidated using coculture of M2 macrophages and Transwell assays *in vitro* and *in vivo* in nude mice and NOD-SCID mice.

**Results:** We demonstrated for the first time that SNAIL and CXCL2 were abnormally overexpressed in colorectal cancer, especially lung metastasis, and were associated with poor prognosis in colorectal cancer patients. We demonstrated that SNAIL promoted the secretion of CXCL2 by mesenchymal cells and induced the activation of M2 macrophages. We found that CXCL2 attracted M2-type macrophages to infiltrate and promote tumour metastasis.

**Conclusion:** These findings suggest that SNAIL promotes epithelial tumour transformation, and that transformed mesenchymal cells secrete CXCL2, which promotes M2 macrophage infiltration and tumour cell metastasis. These findings elucidate the tumour-TAM interaction in the metastatic microenvironment, which is mediated by tumour-derived CXCL2 and affects lung metastasis. This study also provides a theoretical basis for the occurrence of secondary lung cancer.

## Introduction

Colorectal cancer (CRC) is a leading cause of cancer death in the United States, with an estimated 53,000 related deaths in 2020 1. Colorectal cancer (CRC) is the third most common cancer worldwide and the second most common cause of cancer death, with an estimated 1.8 million new cancers diagnosed and 880,000 deaths in 2018 2. These differences may be due to differences in genetic susceptibility, socioeconomic status, environmental exposure, diet, other lifestyle factors and/or screening practices 3. These events cause histological and morphological changes that lead to the rapid spread of cancer to lymph nodes and adjacent or distant organs, especially the lungs, in the most advanced stages of its development 4. Approximately 35% of patients present with metastatic disease at diagnosis, and up to 50% of patients with nonmetastatic colorectal cancer eventually develop metastatic disease 5. An increasing number of studies showed that the tumour microenvironment played an important role in mediating the distant metastasis of colorectal cancer 6. Tumour-associated macrophages (TAMs) play the main role of regulating various factors in the tumour microenvironment and promoting tumour development 7. Lan et al. found that M2-type immunomacrophages promoted the migration and proliferation of tumour cells 8.

However, the tumour microenvironment is complex, and the causes of M2 macrophage infiltration are not known. We analysed the sequencing results of lung metastasis tissue and *in situ* tumour tissue of colorectal cancer and found that EMT was an important cause of macrophage infiltration. Conditioned media from mesenchymal breast cancer cell lines activate macrophages to a TAM-like phenotype, including high expression of CD206 (also known as macrophage glycogen receptor or MMR) and high secretion of CCL17, CCL18, CCL22, and IL-10 9. This finding suggests that tumour stromal cells secrete chemokines that attract macrophage infiltration and mediate tumour progression. Our sequencing results demonstrated that CXCL2 was an important mediating factor in this reaction. However, the factors mediating tumour development of EMT and the occurrence of these processes are not known. Our results showed that SNAIL may be the main cause. Many studies found that SNAIL played an important role in the occurrence and development of EMT, but few studies were performed in colorectal cancer 10.

To further clarify the mechanisms leading to lung metastasis and EMT macrophage infiltration, we investigated the causes of macrophage infiltration that lead to poor prognosis of lung metastasis using macrophage coculture *in vitro* and BALB/c nude and NOD-SCID mice *in vivo*.

## Materials and Methods

### Patients and tissue samples

Thirty-five colorectal cancer specimens with lung metastasis were collected from the colorectal cancer database and tissue bank of the First Affiliated Hospital of Jinan University and the Second People's Hospital of Guangdong Province. Tissue was taken from patients who underwent bowel cancer surgery between 2016 and 2019 without chemotherapy or radiation before surgery. The studies were performed in accordance with the International Code of Ethics for Biomedical Research involving humans (CIOMS). The studies were performed after approval by the Institutional Review Board. We obtained written consent from the subjects before the study was performed.

### Cell culture and treatment

Two human colorectal cancer cell lines (DLD1 and RKO), the murine colon cancer cell line MC38 and the human monocyte cell line Thp-1 were purchased from ATCC. All cell lines were acquired between 2018 and 2020 and cultured according to the ATCC recommended guidelines we described previously 11. The cells were free of mycoplasma and passaged no more than 18 to 25 times after thawing. Thp-1 cells were treated with 200 nmol/L PhorBOL 12-MyriSTATE 13-acetate (Sigma) for 24 h to differentiate into adherent macrophages. Macrophages (5×105) for the coculture experiment were placed in the lower chamber of the 12-well plate, and colorectal cancer cells (5×105) were added to the upper chamber of the 0.4-mm diameter Transwell insert (Millipore).

### Construction of SNAIL and CXCL2 Stable Knockdown and Overexpression Cells

Lentiviruses expressing SNAIL or CXCL2 and lentiviruses expressing negative control shRNA (pLKO) and overexpression control lentiviruses were purchased from GenePharma (Shanghai, China). DLD-1, RKO and MC38 cells were infected with control virus and SNAIL-overexpressing virus (the number of infections was 50). Cells were cultured in the presence of puromycin for 3-4 weeks, and SNAIL levels were detected using WB. To verify the knockdown virus, we purchased three pairs of knockdown plasmids and knocked down SNAIL and CXCL2. The plasmid with the highest knockdown efficiency was selected for subsequent experiments. We constructed SNAIL knockdown-CXCL2 overexpression, SNAIL knockdown-CXCl2 overexpression, SNAIL knockdown-CXCL2 overexpression and PLKO-Con stable cells. We tested it with the protein (Figure S1 A-G).

### Quantitative Reverse Transcription PCR and transcriptome sequencing

Total RNA was extracted from tumour and normal tissues using TRIzol reagent (Ambion, Austin, TX, USA). After reverse transcription using cDNA as a template, mRNA levels were measured using TB Green® Premix Ex Taq™ II (Invitrogen) in the ABI PRISM 7500 system (Applied Biosystems, Foster City, CA, USA). The level of circRNA expression was normalized to glyceraldehyde-3-phosphate dehydrogenase (GAPDH). mRNA expression levels were normalized using U6. The primers used are listed in Schedule S1. Novo Source performed transcriptome sequencing. Colorectal carcinoma *in situ* and lung metastases with 10 samples were selected for sequencing.

### Transwell Assays

Transwell assays were used to detect the migration and invasion of DLD-1 and RKO cells. During the migration experiment, cells in each group were digested with trypsin and washed with serum-free medium three times. Cell microspheres were suspended in serum-free medium, and 100 μL of cell suspension (10000 cells per well) was added to the upper chamber. Medium (600 μL) containing 20% foetal bovine serum was added to the lower chamber. After incubation at 37 °C for 24 h, the Transwell chamber was rinsed twice with PBS and fixed with 5% pentanediol at 4 °C. The cells were stained with a 0.1% crystal violet solution for 10 minutes. After washing with PBS 2 times, the cells at the top of the filter membrane were removed with cotton swabs. A 200-magnification DMi8 microscope (Leica, Wetzlar, Germany) was used to monitor and photograph migrating or invading cells at the bottom of the membrane. Six randomly selected fields were counted. A Transwell chamber with precoated substrate was used in the invasion experiment, and the procedure was the same as the migration experiment. Each experiment was repeated three times. The average number of cells in each field was used as the number of migrated or invaded cells.

### Clone formation experiment on plate

A cell suspension was prepared using conventional digestion and passage methods for exponential cell growth. The cell suspension was repeatedly mixed to fully disperse the cells, and the percentage of single cells was greater than 95%. The cells were counted, the cell concentration was adjusted with medium, and the cells were set aside. The cell suspensions were diluted in multiple proportions according to the cell proliferation capacity. Five millilitres of cell suspension was inoculated into a six-well plate (60 mm in diameter) at concentrations of 50, 100 and 200 cells in each well, and the dish was gently shaken to evenly disperse the cells. Petri dishes were incubated at 37 °C and 5% CO2 for 2-3 weeks, and fresh culture medium was replaced in a timely manner according to pH changes of the culture medium. When visible clones appeared in the dish, the culture was terminated, and the culture medium was discarded. PBS solution was carefully added twice, and the cells were air dried. The cells were fixed with methanol for 15 minutes and air dried after the methanol was discarded. The cells were dyed with Giemsa solution for 10 minutes. The solution was slowly washed away with running water, and the cells were air dried.

### Western blotting

Total protein was extracted using RIPA lysis buffer, and sodium dodecyl sulphate-polyacrylamide gel electrophoresis (SDS-PAGE) was performed. These proteins were transferred to a polyvinylidene fluoride membrane. The membranes were blocked with 5% skim milk and incubated with primary antibodies (anti-SNAIL, anti-CXCL2 and anti-β-actin antibodies). After incubation with secondary antibodies, protein visualisation was performed using SuperSignal West Pico Plus (Thermo Fisher Scientific, Waltham, MA, United States). 1. Anti-SNAIL antibody (AB203225, 1:800, Item no. Ab203225) was purchased from Abcam. The antibody source was rabbit, and the company recommended a Western blot (WB) concentration range of 1:500-1:2000. 2. The anti-CXCL2 antibody (art. PA588673, 100 µl) was purchased from Thermo Fisher Scientific. The source was rabbit, and a Western blot (WB) concentration range of 1:500 to 1:2000 was recommended. 3. The β-actin antibody (art. 4967S, 100 µl) was purchased from Cell Signaling Technology (CST), and the source was rabbit. The recommended Western blot concentration was in the 1:1000 range. 4. The anti-rabbit IgG (Art. 7074S, 1 mL) was purchased from Cell Signaling Technology (CST), and the source was a goat/rabbit antibody with a recommended dilution range of 1:1000 to 1:3000.

### Isolation of M2 macrophages using FACS

The FACS procedure was described in the previous literature. The cells were digested using trypsin and passed through a cell filter (100, 70, 40 µm) 12. CD163 expression was first classified using a CD163 antibody in macrophages differentiated into adherent cells. The isolated cells were continued in culture. CD163 rabbit polyclonal GB113109 (100 µl) was purchased from Wuhan Xaviel Biotechnology Co., Ltd. The recommended concentrations were 1:100 and 1:200, and the company experimental concentration was 1:300.

### Subcutaneous Tumour Models in Nude Mice and NOD-SCID Mice

Female nude mice aged 4-6 weeks were collected from Beijing Weishenghe Experimental Animal Science and Technology Co., Ltd. (Beijing, China). Six nude mice were divided into two groups (n = 3) and subcutaneously injected with MC38 cells or control cells with stable expression of 5 × 10^6^ SNAIL. Six female NOD-SCID mice aged 4-6 weeks were divided into 2 groups (n = 3) and subcutaneously injected with MC38 cells or control cells with stable expression of 5 × 10^6^ SNAIL. Thirty nude mice were divided into 5 groups (n = 6) and subcutaneously injected with 5 × 10^6^ SNAIL-knockdown CXCL2-overexpression, SNAIL-overexpression CXCL2-overexpression, SNAIL-overexpression CXCL2-knockdown, SNAIL-knockdown CXCL2-knockdown and PLKO-Con mutant cells. Tumour volume was measured 30 days after injection. All laboratory procedures involving animals were approved by the Animal Care and Use Committee of Guangdong Second People's Hospital Institution in accordance with the Guidelines for The Care and Use of Laboratory Animals published by the National Institutes of Health (NIH Publication No. 85-23, revised 1996). These experiments were repeated three times.

### Construction of an animal model of lung metastasis

Ten nude mice were divided into 2 groups (n = 6), and 2 × 10^6^ MC38 cells stably expressing SNAIL or control cells were injected through the tail vein. Thirty nude mice were divided into 5 groups (n = 6) and injected with 2 × 10^6^ SNAIL-knockdown CXCL2-overexpression, SNAIL-overexpression CXCL2-overexpression, SNAIL-overexpression CXCL2-knockdown, SNAIL-knockdown CXCL2-knockdown and PLKO-Con mutant cells. The number of lung tumours was measured 40 days after injection. These experiments were repeated three times.

### Immunohistochemistry and immunofluorescence analysis

All paraffin-embedded tissue samples were assayed using streptavidin peroxidase, as described previously 13. Tissue sections and a CXCL2 antibody (1:200; 1:400; 1:600, Thermo Fisher Scientific, MA, USA), anti-Ki67 antibody (1:500; 1:1500, Abcam, Cambridge, UK), anti-vimentin antibody (1:500; 1:800; 1:1000, Wuhan Xavier Biotechnology Co., LTD., Wuhan, China), or anti-e-cadherin antibody (1:500; 1:800; 1:1000, Wuhan Xavier Biotechnology Co., LTD., Wuhan, China) were incubated overnight at 4 °C. The scoring system was established as described above. High and low expression levels of CXCL2, anti-human Ki67, VIM, and ECAD were determined according to the scoring system. For immunofluorescence costaining, a TSAPLus triple fluorescent staining kit (Wuhan Xaviel Biotechnology Co., LTD., Wuhan, China) was used. The tissues were incubated with primary antibodies against CXCL2 and CD163 at 4 °C overnight. The dish was washed with PBS, and the secondary antibody (HRP-labelled goat anti-rabbit IgG, 1:200, Wuhan Xaviel Biotechnology Co., LTD., Wuhan, China) was added and incubated at room temperature for 50 min. The cells were incubated with DAPI (Wuhan Xavier Biotechnology Co., LTD., Wuhan, China) in the dark at room temperature for 10 min. Images were obtained using a Panoramic DESK, P-MIDI, P250 slice digital scanner (3D HISTECH, Hungary). The antibody information is as follows:

### Data preprocessing and DEG identification

The 'limma' R/Bioconductor package was used for preprocessing, including quality control and background correction of all raw expression data from GSE41258 and conversion from probes to gene symbols 14. The conversion from probes to gene symbols removed or averaged probes that were not expressed or genes with more than one probe. The "limma" R package was also used to screen differentially expressed genes (DEGs) between primary colon tumour (186 samples) and normal colon tissue (54 samples) and lung metastasis tissue (20 samples) compared to normal lung tissue (7 samples). A false discovery rate (FDR)<0.05 and |log2FC| ≥1 were set as the criteria for screening DEGs.

### Establishment of weighted correlation network analysis

The coexpression relationship based on gene expression was investigated using the WGCNA package in R 3.6.2 15. Because genes with low expression or unchanged expression generally represent noise, genes were filtered by variance, and the top 20% expressed genes were selected for subsequent analysis. The function "good Samples Genes" of the WGCNA package was first used to assess the input genes and samples. The construction of a weighted gene network requires the selection of the coexpressed soft threshold capability β to improve the similarity and calculate the adjacency. The β value was selected according to the function "pickSoftThreshold", which performed network topology analysis and helped select the appropriate soft threshold based on the standard of approximate scale-free topology. The adjacency matrix was converted to a topological overlap matrix (TOM). TOM measured the network connectivity of a gene, which was defined as the sum of its adjacency with all other genes and used for network generation. To classify genes with similar expression patterns into gene modules, average linkage hierarchical clustering was performed according to the TOM-based dissimilarity measure. The modules' gene expression and external clinical characteristics were subjected to correlation analysis, and intramodular analysis was performed to determine whether the gene expression and clinical correlation were consistent.

### Functional enrichment

Functional enrichment analysis was performed based on the Metascape platform 16, including Gene Ontology (GO) and Kyoto Encyclopedia of Genes and Genomes (KEGG) 17. The background gene for enrichment analysis uses all of the genes in the genome. Collected terms with a P value<0.05, containing at least 3 genes, and enrichment factor>1.5 were grouped according to the similarity of their members. The p value was calculated based on the cumulative hypergeometric distribution, and the q-value was calculated based on the Benjamini-Hochberg (BH) procedure 18.

### Protein-protein Interaction network creation and hub gene identification

For candidate genes, protein-protein interaction enrichment analysis was performed using the STRING database based on the Metascape platform 19. The resulting network contained at least one subset of proteins whose other members formed a physical interaction, and the Molecular Complexity Detection (MCODE) algorithm was applied to further identify densely connected network components 20.

### Evaluation of immune cell infiltration

We used the CIBERSORT method to analyse the significant differential expression of different immune cell types between groups 21. The format of the gene expression profile of lung metastasis tissue and normal lung tissue was preprocessed and prepared according to the format accepted by CIBERSORT. We used the CIBERSORT original gene signature file LM22, which defined 22 immune cell subtypes. For the difference analysis between lung metastasis tissue and the control group, the P value was calculated using the Wilcoxon rank sum test.

### Statistical analysis

SPSS 18.0 software was used for all statistical analyses, and GraphPad Prism 5.0 software was used for plotting. P < 0.05 was considered statistically significant.

## Results

### EMT and M2-type macrophage infiltration are important features of lung metastases

**Figure 1:**
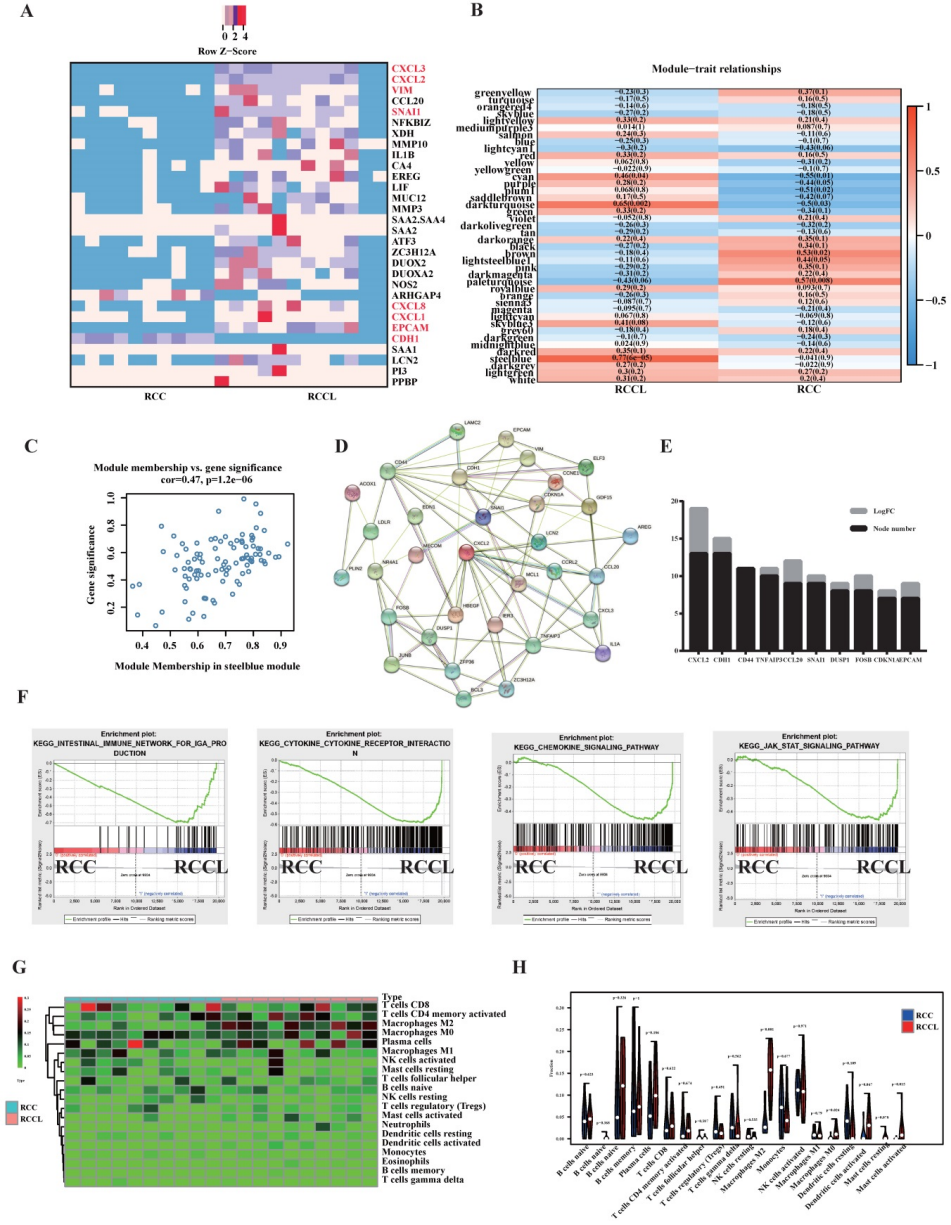
** SNAIL-dominated EMT and CXCL2-dominated macrophage infiltration are important features of lung metastases. (A)** Colorectal carcinoma *in situ* and lung metastases heatmap of the 30 DEGs with |log2fold change | > 2, P < .05. Red: higher expression; blue: lower expression.** (B)** Colorectal carcinoma *in situ* and lung metastases module-trait associations. Each row corresponds to a module eigengene, and each column corresponds to a trait. Each cell contains the corresponding correlation and p value. The table is colour-coded by correlation according to the colour legend. **(C)** A scatter plot of gene significance (GS) and module members (MM) of relapses in the SteelBlue module.** (D)** Modular analysis of PPI networks in lung metastases. **(E)** Module analysis and statistical node number of the PPI network of lung metastatic tumours. **(F)** Pathways enriched by lung metastases, including the Intestinal Immune Network For IGA Production, Cytokine Receptor Interaction, Chemokine Signalling Pathway, and JAK STAT Signalling Pathway.** (G)** Heatmap of immune cell infiltration in lung metastases versus primary tumours.** (H)** Violin diagram of immune cell infiltration in lung metastases versus primary tumours.

To study the difference in gene expression between *in situ* tumours and lung metastatic tumours, we collected clinical colorectal cancer *in situ* tumours and corresponding metastatic tumours for transcriptome sequencing. Heatmaps of the top 30 differentially expressed genes revealed significant changes in EMT-related markers of lung metastasis, such as VIM, CDH1 and SNAIL (see Figure 1A). We also found significant differences in the expression of immune-related indicators, such as CXCL3, CXCL2, CXCL8, and CXCL1 (see Figure 1A). To identify its core genes, we performed calculations using the WGCNA method (see Figure. 1B, C and S2A-F) and concluded that the SteelBlue module was an important gene set affecting lung metastatic tumours (see Figure 1B, C). We further screened core genes using the protein interaction network of the STRING database and found that CXCL2 was an important core gene of lung metastatic tumours (see Figure 1D, E). Enrichment analysis found that lung metastases significantly correlated with the immune microenvironment and chemokine-related pathways and EMT proliferation-related pathways (see Figure 1F). Because lung metastatic tumours have many immune-related pathways and targets, we analysed the immune cell infiltration of lung metastatic tumours and identified the most common infiltrating immune cells. Our analysis found that M2-type macrophages were closely related to lung metastatic tumours (see Figure 1G, H).

To further validate our results, we performed bioinformatics analysis of GEO colorectal carcinoma *in situ* and lung metastatic carcinoma and found that the infiltration of M2-type macrophages in lung metastatic carcinoma (Figure S3A). These studies identified CXCL2 as a core gene of lung metastatic tumours. We reanalysed the results of the public database and found that the expression of CXCL2 in lung metastatic tumours was significantly higher than the *in situ* tumours (see Figure S3B). CXCL2 expression was also significantly higher in tumours than adjacent tissues (see Figure S3C).

### Lung metastases are a group of tumour mesenchymal cells that secrete CXCL2 and attract M2 macrophage infiltration

**Figure 2:**
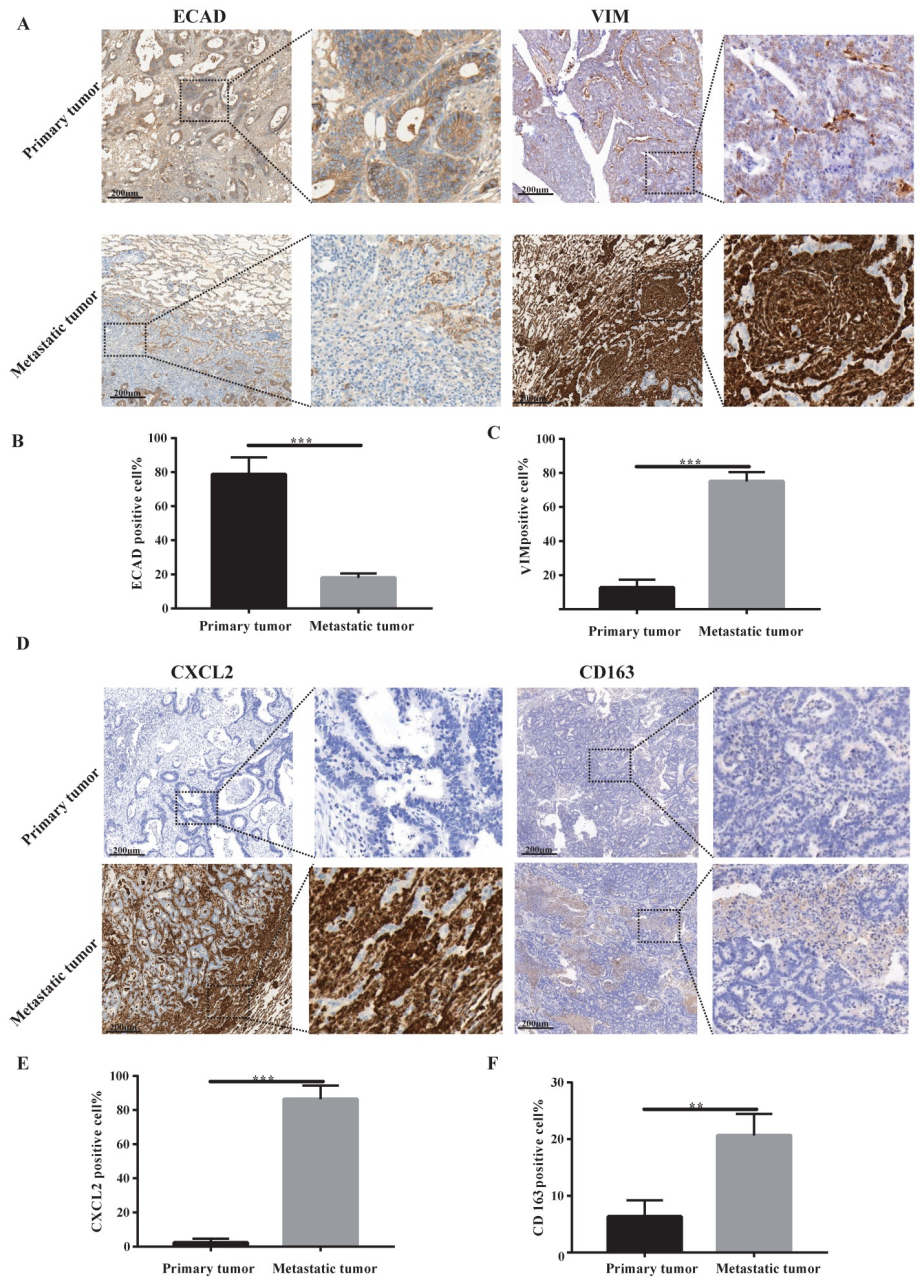
** Lung metastases have prominent mesenchymal features and secrete CXCL2 and M2 macrophage infiltrates. (A)** ECAD and VIM immunohistochemical staining of lung metastases and primary tumours. The scale is 200 µm.** (B, C)** The proportion of positive cells per 100 cells, statistical number of A, ***, P < 0.001.** (D)** Immunohistochemical depictions of CXCL2 and CD163 in lung metastases and primary tumours. The scale is 200 µm.** (E, F)** The proportion of positive cells per 100 cells, statistical number of D, ***, P < 0.001; **, P < 0.01.

Our sequencing results found that pulmonary metastatic tumours exhibited obvious characteristics of EMT and M2-type macrophage infiltration. We analysed whether lung metastases and *in situ* tumours had mesenchymal tumour cell characteristics using immunohistochemistry. According to Figure 2A-C, the stromal cell marker VIM of lung metastatic tumours was significantly higher than the *in situ* tumours, and the epithelial cell marker ECAD was significantly lower than the *in situ* tumours. These findings indicate a large number of tumour stromal cells in lung metastases. We examined certain characteristics of lung metastatic tumour cells. We found that lung metastatic tumours secreted significant CXCL2 chemokines and were infiltrated by a large number of M2-type macrophages (see Figure 2D-F).

### SNAIL induces pulmonary metastasis by mediating EMT

**Figure 3:**
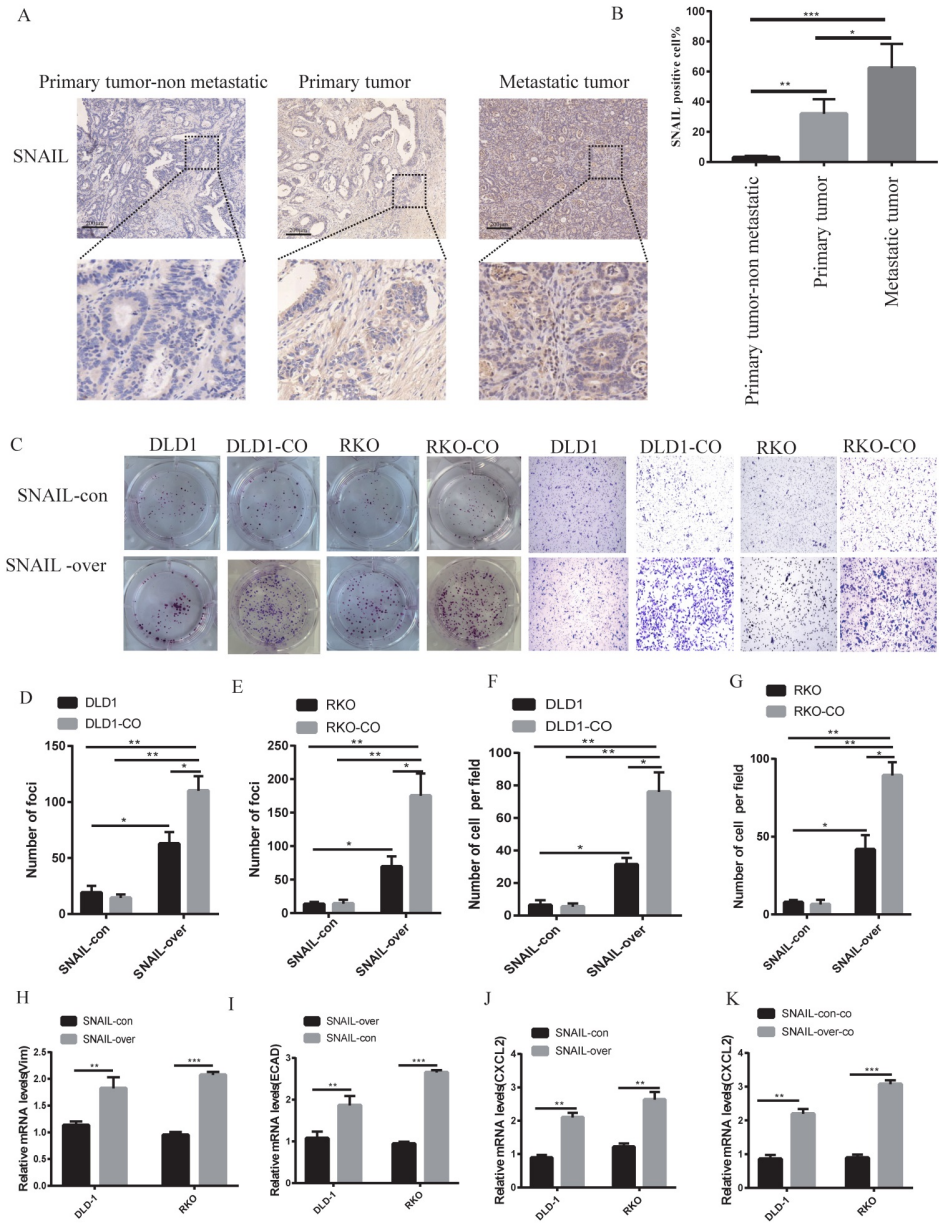
** SNAIL expression is closely related to macrophage promotion of tumour cell migration and metastasis. (A)** Immunohistochemical staining of SNAIL in primary tumours with or without CRC lung metastases, and lung metastases. The scale is 200 µm.** (B)** The proportion of positive cells per 100 cells, statistical number of A, ***, P < 0.001; **, P < 0.01; *, P < 0.05.** (C)** SNAIL overexpression and M2 macrophage coculture for cell proliferation and migration.** (D-G)** C Statistics of colony number and migrating cell number, **, P < 0.01; *, P < 0.05.** (H-I)** Effect of SNAIL overexpression on VIM and ECAD expression in CRC cells, **, P < 0.01. **(J)** Effect of SNAIL overexpression on CXCL2 expression in CRC cells, ***, P < 0.001. **(K)** Effect of SNAIL overexpression on CXCL2 expression in CRC cells co-cultured with TAMs, ***, P < 0.001.

Our previous study showed that lung metastases were a group of mesenchymal tumour cells that secreted CXCL2 to attract M2 macrophages. However, the causes of EMT in lung metastases were not clear. Our sequencing results found that lung metastatic tumour tissue highly expressed SNAIL, and numerous studies showed that SNAIL was an important cause of EMT 22, 23. We analysed SNAIL expression in patients with nonmetastatic colorectal cancer and patients with lung metastasis. SNAIL expression in patients with lung metastasis was significantly higher than patients with nonmetastatic colorectal cancer (see Figure 3A, B). We compared the changes in SNAIL *in situ* tumours and lung metastatic tumours and found that SNAIL expression in lung metastatic tumours was significantly higher than *in situ* tumours (Figure 3A, B).

### SNAIL is an important factor mediating lung metastasis

The factors underlying the effects of SNAIL on migration and proliferation were not known. Colony and migration experiments were performed by overexpressing SNAIL *in vitro*. Although SNAIL overexpression affected tumour migration and proliferation, the migration and proliferation of non-cocultured cells was significantly lower than cocultured M2 macrophages (see Figure 3C-G). SNAIL-overexpressing cells showed obvious features of mesenchymal cells (see Figure 3H-I). SNAIL overexpression led to significant CXCL2 expression (see Figure 3J) but not macrophage production (see Figure 3K). These experiments demonstrated that SNAIL mediated the occurrence of EMT, and SNAIL-induced tumour mesenchymal cells interacted with M2 macrophages to promote the invasion and proliferation of tumour cells.

### SNAIL overexpression promotes EMT and leads to M2 macrophage infiltration and promotion of tumour progression

**Figure 4:**
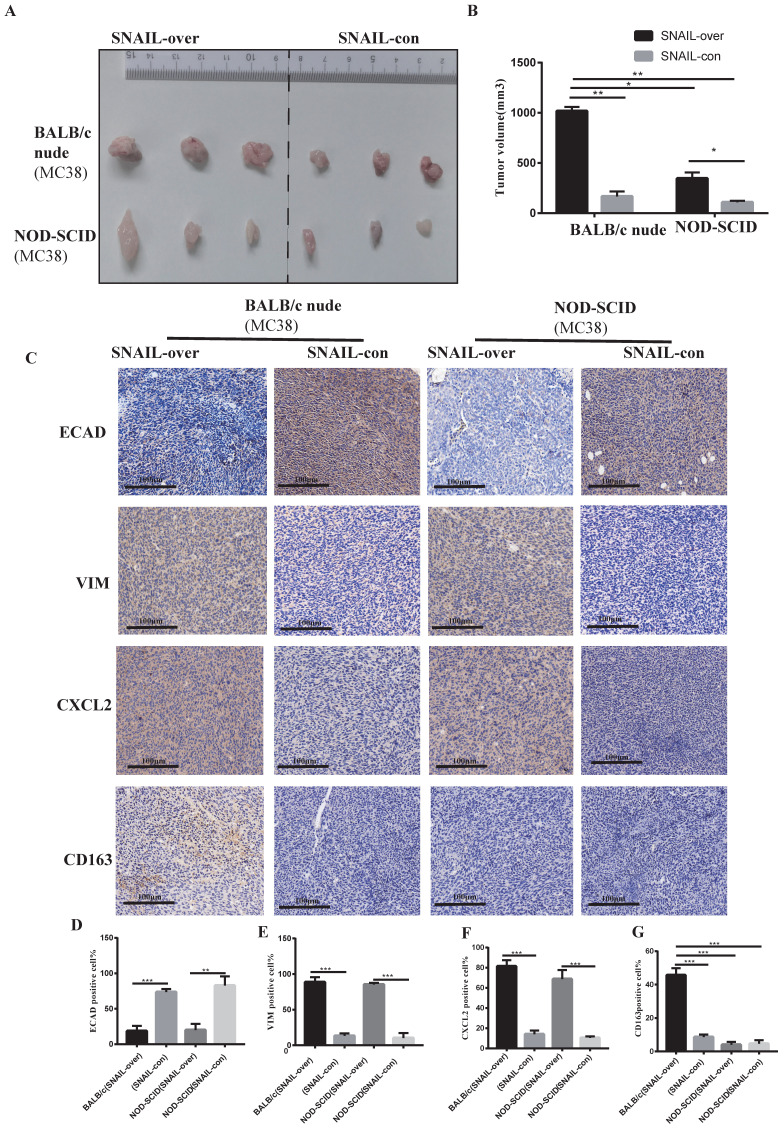
** High SNAIL expression promotes the secretion of CXCL2 by tumour cells after the tumour stroma activated M2 macrophages and induced the migration and proliferation of tumour cells. (A)** SNAIL overexpression in BALB/c nude mice and NOD-SCID mice.** (B)** Statistical comparison of tumour volume after tumour formation, **, P < 0.01; *, P < 0.05.** (C)** Representative diagram of ECAD, VIM, CXCL2 and CD163 immunohistochemical staining in different treatment groups.** (D-G)** The proportion of positive cells per 100 cells, statistical number of C, ***, P < 0.001; **, P < 0.01.

Based on previous studies, we preliminarily found that SNAIL promoted EMT transformation, and transformed mesenchymal tumour cells mediated M2 macrophages to affect tumour migration and proliferation by secreting CXCL2. However, whether CXCL2 attracted macrophage infiltration via secretion was not known. Therefore, we studied the effects of macrophages on tumours in BALB/c nude mice and NOD-SCID mice. Because macrophages in BALB/C nude mice are normal compared to NOD-SCID mice, these models allowed us to study the effects of macrophages on tumours. The SNAIL overexpression and control groups were subcutaneously injected into BALB/C nude mice and NOD-SCID mice, and the tumour size of the SNAIL overexpression group was significantly larger than the control group (see Figure 4A, B). However, SNAIL overexpression was significantly higher in BALB/c nude mice than NOD-SCID mice (see Figure 4A, B). In conclusion, SNAIL overexpression was more conducive to tumour proliferation in mice with normal macrophages. We analysed SNAIL overexpression in EMT and macrophages from the above two groups of mice and CXCL2. We found that SNAIL overexpression promoted the EMT of tumours (see Figure 4C-E) and the expression of CXCL2 (see Figure 4C, F). However, there were significant differences in M2 macrophages, and SNAIL overexpression significantly correlated with macrophage infiltration in BALB/c nude mice (see Figure 4C, G). Therefore, SNAIL enriched the infiltration of macrophages by promoting EMT and CXCL2 secretion, and the infiltration of macrophages was an important factor in promoting tumour proliferation and migration (see Figure S3A, C).

### SNAIL regulates EMT and secretes CXCL2 to attract M2 macrophages and promote proliferation and migration

**Figure 5:**
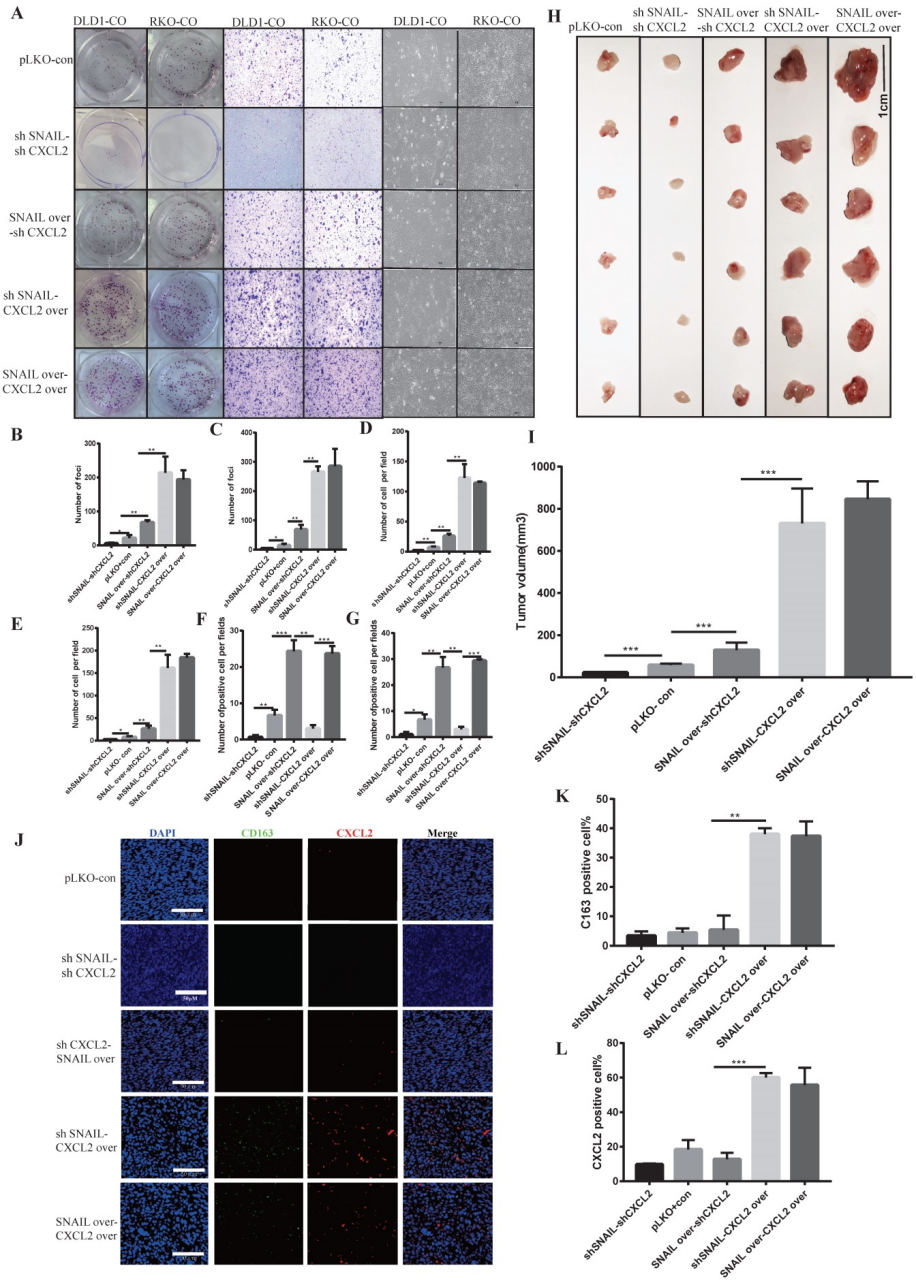
** CXCL2 activates M2 macrophages and induces the migration and proliferation of tumour cells, leading to SNAIL-mediated tumour proliferation. (A)** Effects of SNAIL overexpression or knockout, CXCL2 overexpression or knockout co-culture with TAMs on the ability of proliferation, migration and morphology in CRC cells. **(B)** Number of DLD1 colonies in A, **, P < 0.01; *, P < 0.05.** (C)** Statistics of RKO colony number in A, **, P < 0.01; *, P < 0.05. **(D)** Number of DLD1 migrated cells in A, **, P < 0.01. **(E)** The number of RKO migrating cells in A, **, P < 0.01; *, P < 0.05.** (F)** The number of DLD1 morphology change cells in A, ***, P < 0.001; **, P < 0.01. **(G)** The number of RKO morphology change cells in A, ***, P < 0.001; **, P < 0.01; *, P < 0.05.** (H)** SNAIL and CXCL2 overexpression or knockdown in BALB/c nude mice. **(I)** Statistical comparison of tumour volume after tumour formation, ***, P < 0.001. **(J)** SNAIL and CXCL2 overexpression or knockdown in BALB/c nude mice were subcutaneously examined by tumour immunofluorescence staining, as shown in the representative figure. The scale is 50 µm. **(K)** The proportion of CD163-positive cells per 100 cells, the number of J, **, P < 0.01.** (L)** The proportion of CXCL2-positive cells per 100 cells, statistical number of J, ***, P < 0.001.

Our previous studies found that SNAIL promoted tumour proliferation and migration by affecting tumour EMT. Tumour mesenchymal cells secreted CXCL2 to attract M2 macrophage infiltration and promote the proliferation and migration of tumour cells. To verify whether CXCL2 played a direct role in attracting macrophages, we studied the roles of CXCL2 and SNAIL. We compared CXCL2 knockdown with SNAIL knockdown, CXCL2 knockdown with SNAIL overexpression, CXCL2 overexpression with SNAIL knockdown and CXCL2 overexpression with SNAIL overexpression found that CXCL2 function and expression were sufficient to affect the migration and proliferation of tumours under coculture conditions (see Figure 5A-G). Although SNAIL overexpression was significantly increased compared to the control group, it was weaker than CXCL2 overexpression. Our *in vivo* nude mouse experiment showed the same results (see Figure 5H-I and Figure S4A-D). Although SNAIL affected EMT, the secretion of CXCL2 by mesenchymal tumour cells, rather than SNAIL, attracted macrophages. The immunofluorescence results also showed that only CXCL2 with sufficient function and expression attracted enough macrophages to produce effects (see Figure 5J-L).

### SNAIL secretes CXCL2 by affecting EMT and attracts M2 macrophages to infiltrate and promote lung metastasis

**Figure 6:**
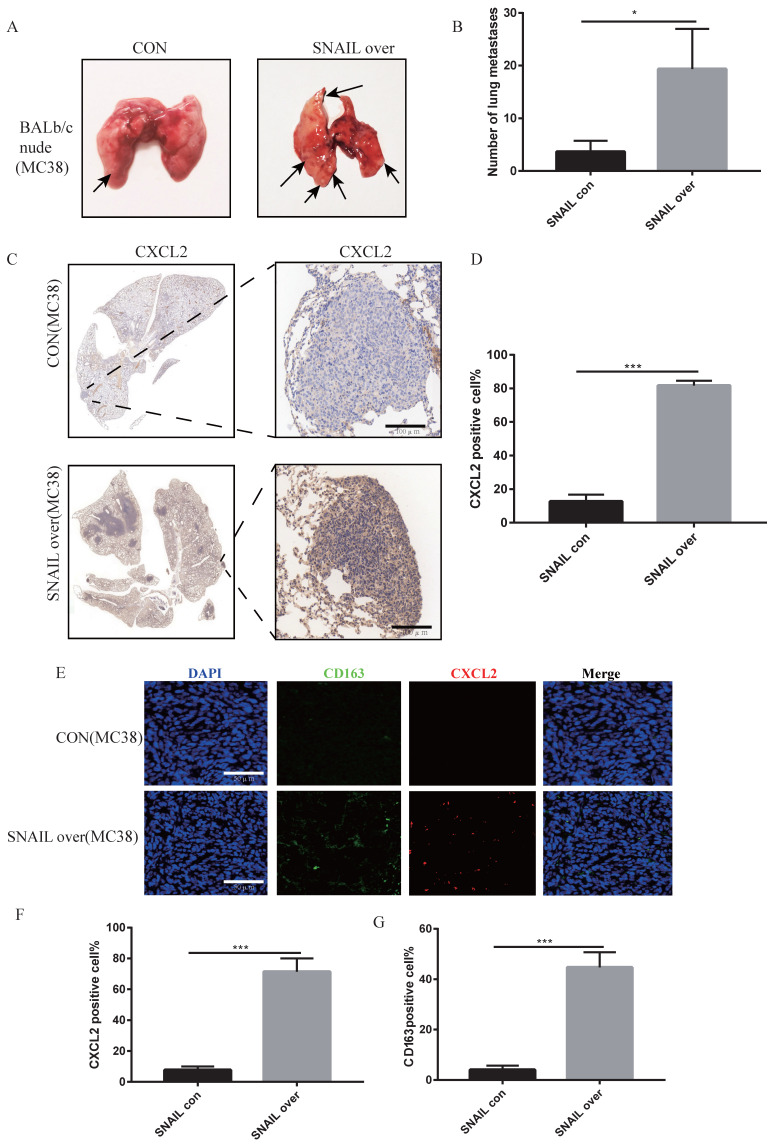
** SNAIL overexpression promotes the secretion of CXCL2 by tumour cells after tumour interstitial activation of M2-type macrophages to induce lung metastasis. (A)** Pulmonary metastasis of the MC38 cell line (overexpressing SNAIL) after tail vein injection in BALB/c nude mice.** (B)** Statistical comparison of the number of lung metastases after tumour formation, *, P < 0.05.** (C)** Immunohistochemical staining of lung metastases in nude mice after tail vein injection of BALB/c nude mice. **(D)** The proportion of CXCL2-positive cells per 100 cells, the number of C, ***, P < 0.001. **(E)** Immunofluorescence staining of lung metastatic tumours after tail vein injection of BALB/c in nude mice. The scale represents 50 µm.** (F)** The proportion of CXCL2-positive cells per 100 cells, statistical number of E, ***, P < 0.001.** (G)** The proportion of CD163-positive cells per 100 cells, statistical number of E, ***, P < 0.001.

We previously demonstrated that SNAIL secreted a large amount of CXCL2 to attract M2 macrophages by affecting EMT, which promoted the migration and proliferation of tumour cells. However, whether these factors affected lung metastasis was not known. SNAIL-overexpressing tumour cells were injected through the caudal vein to analyse the effects of SNAIL on lung metastasis. Our study found that SNAIL overexpression significantly affected the lung metastasis of tumours (Figure 6A-B). Immunohistochemistry showed that SNAIL overexpression affected the occurrence of EMT and CXCL2 secretion (see Figure 6C-D and S5A, B). And the CXCL2 is important to the pulmonary metastasis (see Figure S6A- D). SNAIL overexpression attracted more macrophages by promoting the expression of CXCL2, which led to pulmonary metastasis (see Figure 6E-G, Figure S6E- G).

## Discussion

The current study analysed transcriptome sequencing of tumours *in situ* and metastatic lung tumours and found that EMT and M2 macrophage infiltration were important causes of lung metastasis. We analysed the pathological sections of patients and found that SNAIL promoted EMT, and the transformed mesenchymal tumour cells secreted CXCL2, which promoted the infiltration of macrophages and led to the migration of tumour cells. To compare the role of M2 macrophages in tumours *in vivo*, we used BALB/c nude mice and NOD-SCID mice and found that SNAIL played a role in promoting EMT. Mesenchymal tumour cells attracted infiltrating M2 macrophages by secreting CXCL2, which led to tumour invasion, metastasis and proliferation. SNAIL and CXCL2 have different mechanisms in promoting the progression of colorectal cancer and lung metastasis.

Previous studies showed that SNAIL was upregulated during the progression of colorectal cancer in patients and played a role primarily by regulating tumour EMT 24. Epithelial to mesenchymal transformation (EMT) is a complex cellular process in which epithelial cells acquire mesenchymal phenotypes 25. During EMT, tumour cells undergo tight junction lysis, apical-basal polarity disruption, and cytoskeletal restructuring, which lead to an aggressive phenotype. EMT in cancer cells is abnormally regulated by extracellular stimuli from the tumour microenvironment, including growth factors and inflammatory cytokines, as well as physical pressures within the tumour, such as hypoxia 26. Some transcription factors, such as SNAIL, Twist and ZEB1, are e-cadherin suppressors and EMT inducers 27. Notably, these transcription factors are well known for their role in early embryogenesis and tumour metastasis. In general, the expression of the transcription factor SNAIL is critical for mesoderm and neural ridge formation, and it was initially identified in Drosophila as a shotgun suppressor (e-cadherin homologue), which indicates its fundamental role in morphogenesis 28. SNAIL transcription factors (TFs) are direct inhibitors of e-cadherin transcription of the epithelial morphological promoter that drive EMT 29. SNAIL mediates the occurrence and development of EMT in colorectal cancer. Our study showed that SNAIL also affected the invasion and proliferation of tumours. Because SNAIL exerts its migration ability by influencing EMT, we investigated its effects on tumour metastasis, especially lung metastasis. Our study found that SNAIL was an important gene mediating lung metastasis of colorectal cancer.

We also found that M2 macrophages were an important cause of lung metastasis. Macrophages are multifunctional immune cells that perform a wide range of functions, from regulating tissue homeostasis to defending against pathogens and promoting wound healing 30. Tumour-associated macrophages (TAMs) are macrophages that invade tumour tissue or aggregate in the solid tumour microenvironment. TAMs are an important component of the tumour microenvironment and affect tumour growth, tumour angiogenesis, immune regulation, metastasis and chemotherapy resistance. Most TAMs congregate in the anterior and avascular areas, but other TAMs are found along the lumen wall 31. Activated macrophages are generally divided into two phenotypes, M1 (classical activated macrophages) and M2 (substitute activated macrophages) 32. In general, M1 macrophages promote inflammatory responses to invading pathogens and tumour cells, and M2 macrophages tend to exhibit an immunosuppressive phenotype that is conducive to tissue repair and tumour progression. The two types of macrophages express distinct markers and exhibit different metabolic characteristics and gene expression profiles. M1 macrophages secrete proinflammatory cytokines, such as IL-12, tumour necrosis factor-α, CXCL-10, and interferon (IFN)-γ, and produce high levels of nitric oxide synthase (NOS, an enzyme that metabolizes arginine into the "killer" molecule nitric oxide). M2 macrophages secrete anti-inflammatory cytokines, such as IL-10, IL-13 and IL-4, and express abundant arginase-1, mannose receptor (MR, CD206) and scavenger receptor 33. M2 macrophages are closely related to the migration and proliferation of tumours 34. The mechanisms of the effects of M2 macrophages on tumour invasion and migration remain controversial. Wei et al. found that TAMs induced the EMT program to enhance the migration and invasion of CRC and CTC-mediated metastasis by regulating the JAK2/STAT3/miR-506-33p/FoxQ1 axis, which led to the production of CCL2 and promoted the recruitment of macrophages 35. New interactions between immune cells and tumour cells in the CRC microenvironment have been revealed 6. These interactions promote tumour growth by stimulating angiogenesis and inhibiting acquired immune responses. Kitamura et al. found that CCL2-induced chemokine cascades promoted breast cancer metastasis by enhancing the retention of metastasis-related macrophages, which led to tumour cell escape via inhibition of the immune response 36. This class is generally associated with chemokines. Our study found that SNAIL induced the continuous secretion of CXCL2 by promoting the occurrence of EMT, which promoted the enrichment of immunosuppressed macrophages and mediated the immune escape of tumour cells.

In summary, we found a novel interaction between immune cells and tumour cells in the colorectal cancer microenvironment. SNAIL induced EMT. After EMT, tumour mesenchymal cells acted on M2 macrophages and tumour cells by secreting CXCL2 and inducing the migration of tumour cells, which led to lung metastasis. TAM targeting may be a promising therapeutic strategy for improving lung metastasis in colorectal cancer.

## Supplementary Material

Supplementary figures.
