# Supplementary material for: SNAIL Induces EMT and Lung Metastasis of Tumours Secreting CXCL2 to Promote the Invasion of M2-Type Immunosuppressed Macrophages in Colorectal Cancer
Source: Int J Biol Sci. 2022 Apr 11;18(7):2867–81. doi: 10.7150/ijbs.66854 (PMC9066124; doi:10.7150/ijbs.66854)
Supplement: Supplementary file 1 — Supplementary figures. [file ijbsv18p2867s1.pdf]

Figure S1. CXCL2 and SNAIL overexpression or knockdown in WB. (A) Representative WB image of SNAIL overexpression or knockdown and CXCL2 overexpression or knockdown **(B, C)** Protein quantification of A, **, P < 0.01. **(D)** DLD1 and RKO with or without SNAIL overexpression is shown in the WB representative image. **(E)** Protein quantification of D, **, P < 0.01. **(D)** DLD1 and RKO with or without SNAIL overexpression is shown in the WB representative image. **(E)** Protein quantification of D, ***, P < 0.001. **(F)** MC38 with or without SNAIL overexpression is shown in the WB representative image. **(G)** Protein quantification of F, **, P < 0.01.

Figure S2. WGCNA process data. (A) Sample clustering detection outliers. All samples are in the cluster, and all samples made the cut-off. (**B**) Sample clustering tree with characteristic heatmaps. **(C)** Network topology analysis at various soft threshold powers. The left panel shows the scale-free fitting index (Y-axis) as a function of soft threshold power (X-axis). The panel on the right shows average connectivity (degree, Y-axis) as a function of soft threshold power (X-axis). **(D)** Clustering tree of genes with different similarities based on topological overlap and assigned module colours. **(E)** Details of the sample clustering tree. **(F)** The heatmap in the panel shows the intrinsic adjacency relationship.

Figure S3. Changes in CXCL2 and M2 macrophages in colorectal cancer. (A) Comparison of lung metastatic tumour and primary tumour enrichment analysis in the GEO database. **(B)** Comparison of CXCL2 expression levels between lung metastatic tumours and primary tumours

in the GEO database, *, $P < 0.05$. **(C)** Comparison of CXCL2 expression levels between tumours and normal tissue of colon in the TCGA database, *, $P < 0.05$.

Figure S4. Effect of CXCL2 and SNAIL overexpression on tumour proliferation. (A) Immunohistochemical staining of subcutaneous tumour-forming proliferation in BALB/c nude mice and NOD-SCID mice with or without SNAIL overexpression. **(B)** SNAIL overexpression with or without knockdown, CXCL2 overexpression with or without knockdown in subcutaneous tumour-forming proliferation of BALB/c nude mice. **(C)** The proportion of Ki67-positive cells per 100 cells, statistical number of A, ***, $P < 0.001$. **(D)** The proportion of Ki67-positive cells per 100 cells, statistical number of B, **, $P < 0.01$; *, $P < 0.05$.

Figure S5. SNAIL affects the occurrence and development of EMT in lung metastases. (A) Representative diagram of immunohistochemical staining of lung metastatic tumours after tail vein injection of BALB/c nude mice. **(B)** The proportion of VIM-positive cells per 100 cells, statistical number A, ***, $P < 0.001$. **(C)** The proportion of ECAD-positive cells per 100 cells, statistical number of A, ***, $P < 0.001$. **(D)** The proportion of Ki67-positive cells per 100 cells, statistical number of A, ***, $P < 0.001$.

Figure S6. SNAIL and CXCL2 overexpression promote lung metastasis. (A) Pulmonary metastasis of the MC38 cell line after tail vein injection in BALB/c nude mice. **(B)** Statistical comparison of the number of lung metastases after tumour formation, ***, $P < 0.001$. **(C)** Immunohistochemical staining of lung metastases in nude mice after tail vein injection of BALB/c nude mice. The scale is 100 μm. **(D)** The proportion of Ki67-positive cells per 100 cells, statistical

number of C, ***, $P < 0.001$. **(E)** Immunofluorescence staining of lung metastatic tumours after tail vein injection of BALB/c nude mice. The scale represents 50 μm. **(F)** The proportion of CD163-positive cells per 100 cells, statistical number of E, ***, $P < 0.001$. **(G)** The proportion of CXCL2-positive cells per 100 cells, statistical number of E, **, $P < 0.01$.

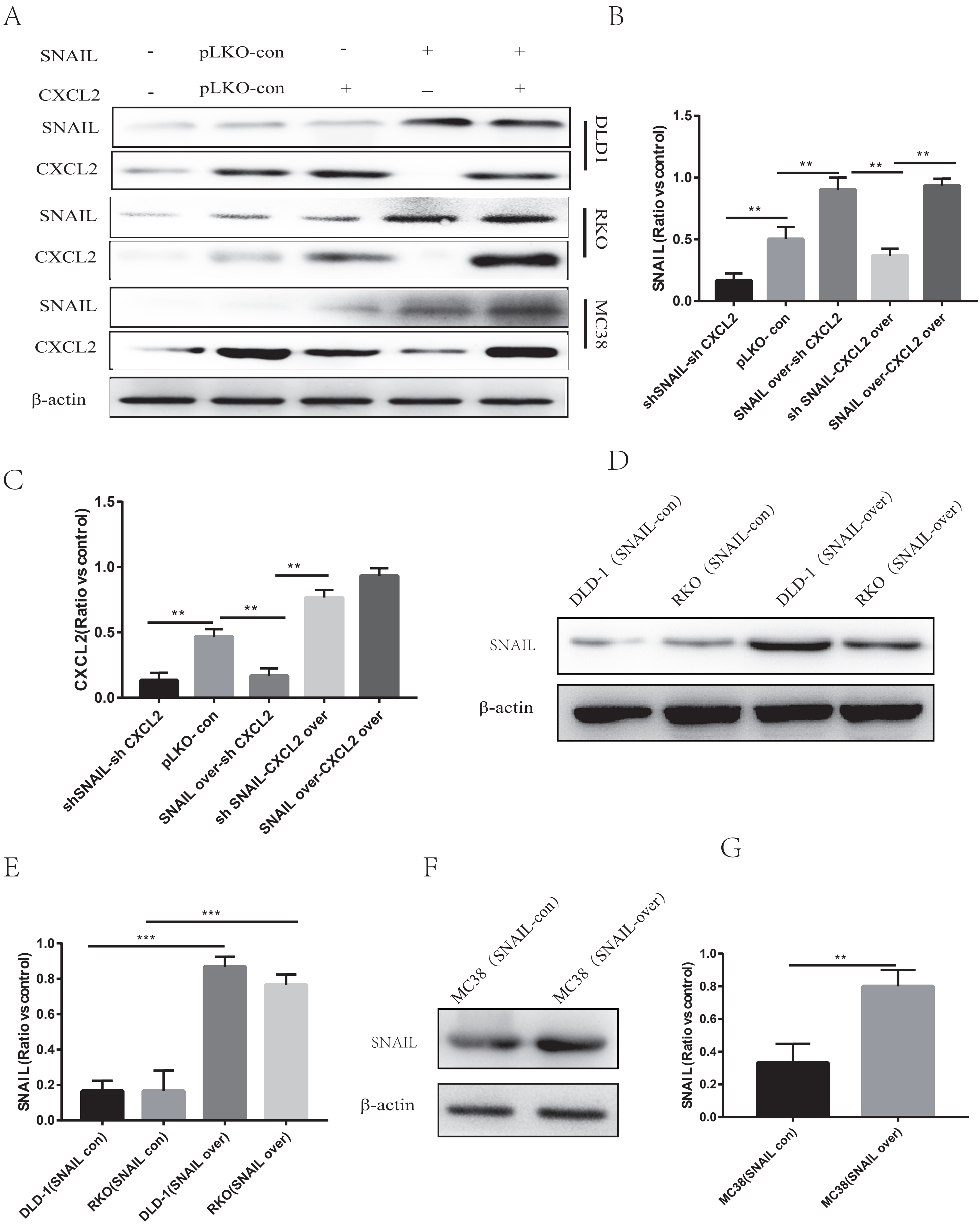
A
SNAIL - pLKO-con - + +
CXCL2 - pLKO-con + – +
SNAIL
CXCL2
SNAIL
CXCL2
SNAIL
CXCL2
β-actin
DLD1
RKO
MC38
B
SNAIL (Ratio vs control)
shSNAIL-sh CXCL2
pLKO- con
SNAIL over-sh CXCL2
sh SNAIL-CXCL2 over
SNAIL over-CXCL2 over
C
CXCL2(Ratio vs control)
shSNAIL-sh CXCL2
pLKO- con
SNAIL over-sh CXCL2
sh SNAIL-CXCL2 over
SNAIL over-CXCL2 over
D
DLD-1（SNAIL-con）
RKO（SNAIL-con）
DLD-1（SNAIL-over）
RKO（SNAIL-over）
SNAIL
β-actin
E
SNAIL (Ratio vs control)
DLD-1(SNAIL con)
RKO(SNAIL con)
DLD-1(SNAIL over)
RKO(SNAIL over)
F
MC38（SNAIL-con）
MC38（SNAIL-over）
SNAIL
β-actin
G
SNAIL (Ratio vs control)
MC38(SNAIL con)
MC38(SNAIL over)

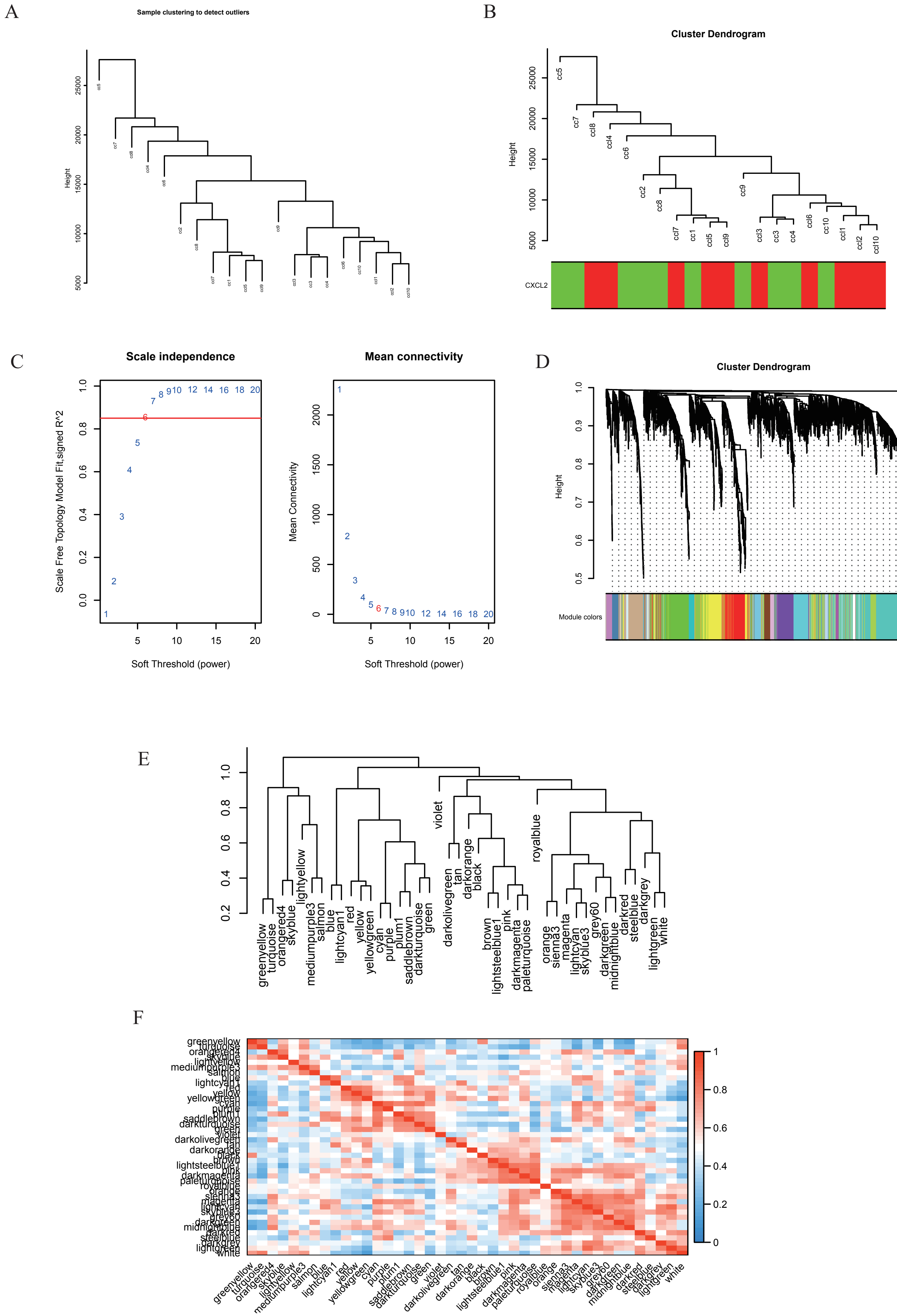
A
Sample clustering to detect outliers
Height
B
Cluster Dendrogram
Height
CXCL2
C
Scale independence
Scale Free Topology Model Fit,signed R^2
Soft Threshold (power)
Mean connectivity
Mean Connectivity
Soft Threshold (power)
D
Cluster Dendrogram
Height
Module colors
E
F

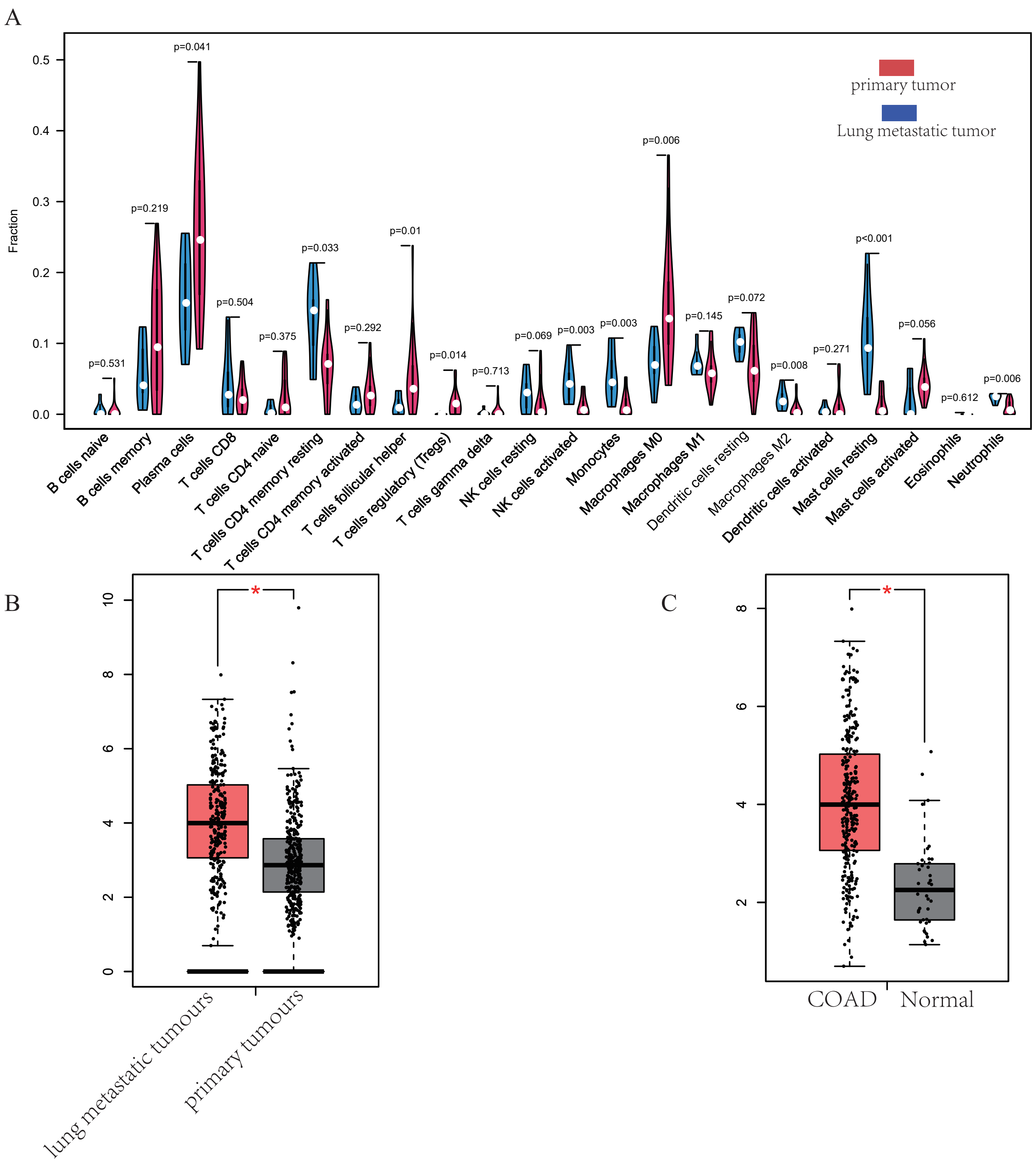

A
primary tumor
Lung metastatic tumor
Fraction
p=0.531
p=0.219
p=0.041
p=0.504
p=0.375
p=0.033
p=0.292
p=0.01
p=0.014
p=0.713
p=0.069
p=0.003
p=0.003
p=0.006
p=0.145
p=0.072
p=0.008
p=0.271
p<0.001
p=0.056
p=0.612
p=0.006
B cells naive
B cells memory
Plasma cells
T cells CD8
T cells CD4 naive
T cells CD4 memory resting
T cells CD4 memory activated
T cells follicular helper
T cells regulatory (Tregs)
T cells gamma delta
NK cells resting
NK cells activated
Monocytes
Macrophages M0
Macrophages M1
Dendritic cells resting
Macrophages M2
Dendritic cells activated
Mast cells resting
Mast cells activated
Eosinophils
Neutrophils
B
lung metastatic tumours
primary tumours
C
COAD
Normal

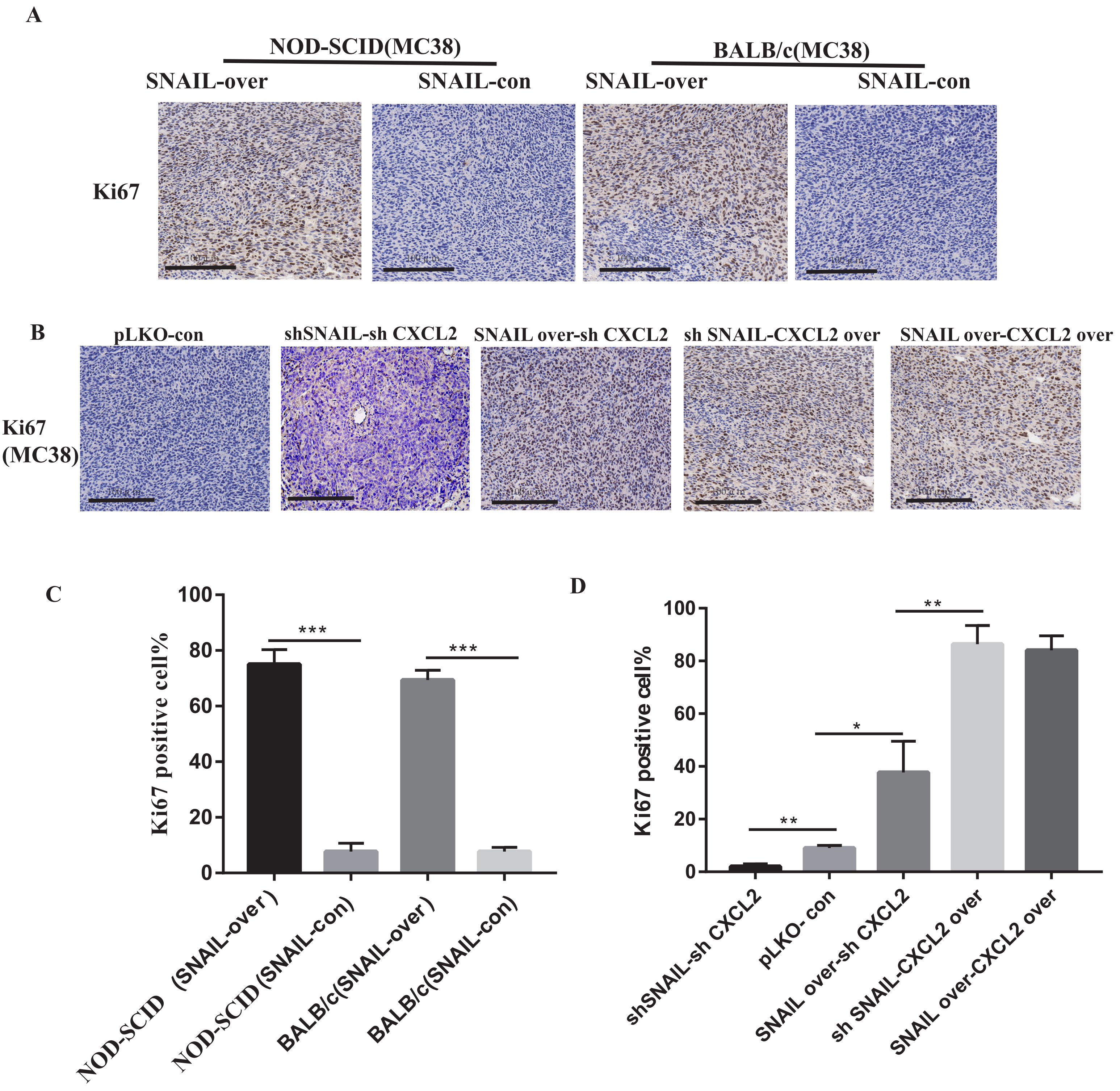
A
NOD-SCID(MC38)
BALB/c(MC38)
SNAIL-over
SNAIL-con
SNAIL-over
SNAIL-con
Ki67
B
pLKO-con
shSNAIL-sh CXCL2
SNAIL over-sh CXCL2
sh SNAIL-CXCL2 over
SNAIL over-CXCL2 over
Ki67
(MC38)
C
Ki67 positive cell%
100
80
60
40
20
0
***
***
NOD-SCID (SNAIL-over)
NOD-SCID (SNAIL-con)
BALB/c(SNAIL-over)
BALB/c(SNAIL-con)
D
Ki67 positive cell%
100
80
60
40
20
0
**
*
**
shSNAIL-sh CXCL2
pLKO- con
SNAIL over-sh CXCL2
sh SNAIL-CXCL2 over
SNAIL over-CXCL2 over

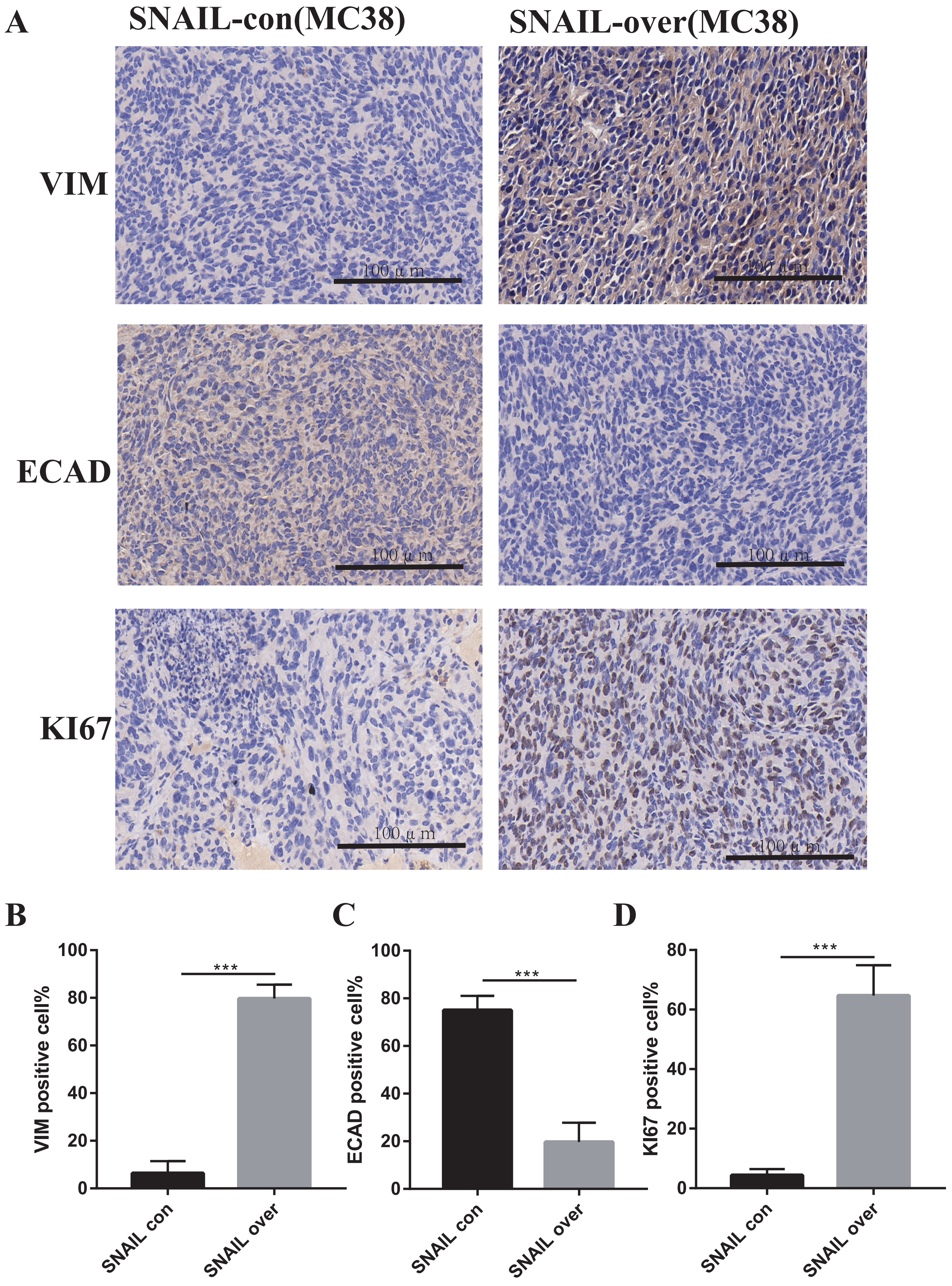
A
SNAIL-con(MC38)
SNAIL-over(MC38)
VIM
ECAD
KI67
100 μ m
B
C
D
VIM positive cell%
ECAD positive cell%
KI67 positive cell%
***
SNAIL con
SNAIL over
0
20
40
60
80
100

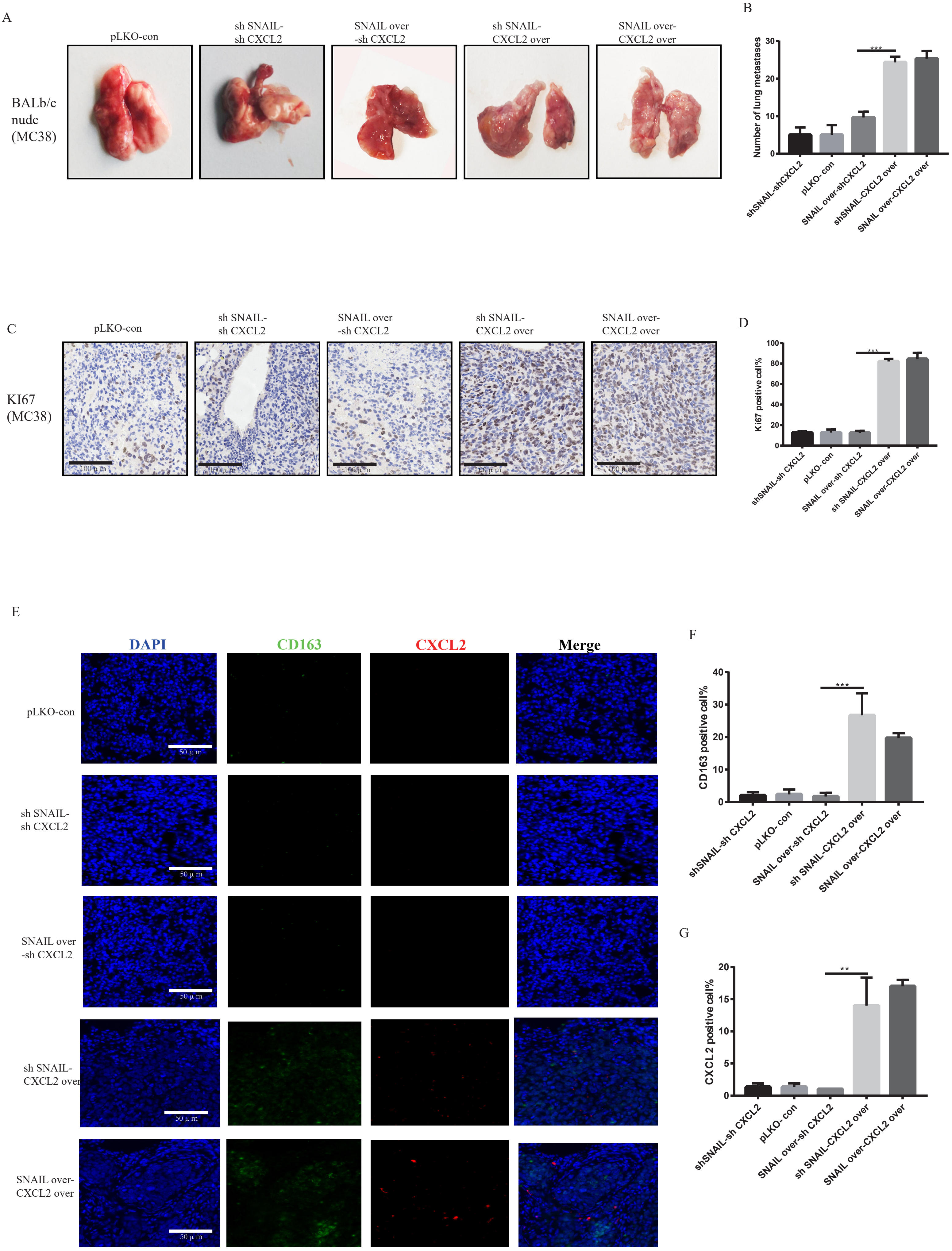
A
pLKO-con
sh SNAIL-
sh CXCL2
SNAIL over
-sh CXCL2
sh SNAIL-
CXCL2 over
SNAIL over-
CXCL2 over
BALb/c
nude
(MC38)
B
Number of lung metastases
***
shSNAIL-shCXCL2
pLKO- con
SNAIL over-shCXCL2
shSNAIL-CXCL2 over
SNAIL over-CXCL2 over
C
KI67
(MC38)
D
Ki67 positive cell%
shSNAIL-sh CXCL2
SNAIL over-sh CXCL2
sh SNAIL-CXCL2 over
E
DAPI
CD163
CXCL2
Merge
50 μ m
F
CD163 positive cell%
G
CXCL2 positive cell%
**
